# Investigating the Influence of Conventional vs. Ultra-High Dose Rate Proton Irradiation Under Normoxic or Hypoxic Conditions on Multiple Developmental Endpoints in Zebrafish Embryos

**DOI:** 10.3390/cancers17152564

**Published:** 2025-08-03

**Authors:** Alessia Faggian, Gaia Pucci, Enrico Verroi, Alberto Fasolini, Stefano Lorentini, Sara Citter, Maria Caterina Mione, Marco Calvaruso, Giorgio Russo, Emanuele Scifoni, Giusi Irma Forte, Francesco Tommasino, Alessandra Bisio

**Affiliations:** 1 Department of Physics, University of Trento, 38123 Trento, Italy; alessia.faggian@unitn.it (A.F.); alberto.fasolini@studenti.unitn.it (A.F.); sara.citter@studenti.unipd.it (S.C.); emanuele.scifoni@tifpa.infn.it (E.S.); 2 Department of Cellular, Computational and Integrative Biology, University of Trento, 38123 Trento, Italy; mariacaterina.mione@unitn.it (M.C.M.); alessandra.bisio@unitn.it (A.B.); 3 Institute of Bioimaging and Complex Biological Systems—National Research Council (IBSBC-CNR), 90015 Cefalù, Italy; gaiapucci@cnr.it (G.P.); marco.calvaruso@cnr.it (M.C.); giorgio-russo@cnr.it (G.R.); giusiirma.forte@cnr.it (G.I.F.); 4 Trento Institute for Fundamental Physics and Applications (TIFPA), Istituto Nazionale Fisica Nucleare (INFN), 38123 Trento, Italy; enrico.verroi@tifpa.infn.it (E.V.); stefano.lorentini@apss.tn.it (S.L.); 5 U.O. Fisica Sanitaria, Therapy Department, Trento Hospital, Azienda Provinciale per i Servizi Sanitari (APSS), 38123 Trento, Italy

**Keywords:** proton therapy, ultra-high dose rate, FLASH effect, zebrafish, radiobiology, radiotherapy

## Abstract

Objectives: To investigate how the FLASH effect modulates radiation response on multiple developmental endpoints of zebrafish embryos under normoxic and hypoxic conditions, after irradiation with proton beams at a conventional and an ultra-high dose rate (UHDR). Methods: Embryos were obtained from adult zebrafish and irradiated with a 228 MeV proton beam 24 h post-fertilization (hpf) at a dose rate of 0.6 and 317 Gy/s. For the hypoxic group, samples were kept inside a hypoxic chamber prior to irradiation, while standard incubation was adopted for the normoxic group. After irradiation, images of single embryos were acquired, and radiation effects on larval length, yolk absorption, pericardial edema, head size, eye size, and spinal curvature were assessed at specific time points. Results: Data indicate a general trend of significantly reduced toxicity after exposure to a UHDR compared to conventional regimes, which is maintained under both normoxic and hypoxic conditions. Differences are significant for the levels of pericardial edema induced by a UHDR versus conventional irradiation in normoxic conditions, and for eye and head size in hypoxic conditions. The toxicity scoring analysis shows a tendency toward a protective effect of the UHDR, which appears to be associated with a lower percentage of embryos in the high score categories. Conclusions: A radioprotective effect at a UHDR is observed both for normoxic (pericardial edema) and hypoxic (head and eye size) conditions. These results suggest that while the UHDR may preserve a potential to reduce radiation-induced damage, its protective effects are endpoint-dependent; the role of oxygenation might also be dependent on the tissue involved.

## Simple Summary

FLASH radiotherapy is based on the selective sparing of normal tissue side effects, keeping the same anti-tumor effectiveness, when the irradiation is performed at an ultra-high dose rate (i.e., >40 Gy/s). In this study, we explore the response of zebrafish embryos after proton irradiation at conventional and ultra-high dose rates, in terms of multiple developmental endpoints. The role of oxygenation at the time of irradiation is also explored. Overall, our data indicate that the so-called FLASH effect can be observed either under normoxic and hypoxic conditions, being more or less pronounced depending on the specific tissue and endpoint considered. This suggests that the sparing effect can have a significant dependence on the specific tissue, and that oxygenation could play a different role depending on the tissue type.

## 1. Introduction

In recent years, a new scenario has been opened in the field of radiotherapy (RT), related to the so-called FLASH effect. This consists of the reduction in normal tissue toxicities while maintaining high anti-tumor efficiency, which arises when a radiation dose is delivered in a very short time (i.e., at an ultra-high dose rate, UHDR) compared to conventional treatments. After the seminal paper by Favaudon and colleagues appeared in 2014 [1], an obvious interest was registered in the RT community. When translated to the clinics, the FLASH effect might provide a unique tool to enlarge the therapeutic window, namely the dosimetric distance between tumor control and normal tissue complication probabilities. The practical implications of this result were soon acknowledged, and extensive research started.

However, all that glitters is not gold. Almost in parallel with the enthusiasm raised around the experimental results, the difficulty to grasp the exact conditions under which the FLASH effect appears and could then be exploited emerged. While independent research groups [2,3], employing different animal models and irradiation approaches, reported results consistent with those obtained by Favaudon et al., there were also a significant number of negative reports, challenging the promising potential of the UHDR.

After about ten years of research on the topic, some results can be considered overall established. First, experimental studies indicate that quite specific irradiation conditions trigger the FLASH effect. Quasi-threshold values have been identified for the dose and dose rates, which are required to be above approximately 8 Gy and 40 Gy/s (i.e., the UHDR), respectively. These values are higher than the 1.8–2 Gy delivered in typical RT treatment at a dose rate of ≅ 0.1 Gy/s. Although studies show that temporal aspects on a smaller scale, associated with the delivery of dose pulses at the micro- or millisecond level, could also play a role, the reported values are typically considered a reference.

From a practical point of view, this implies that the delivery of UHDR treatments would require technical aspects to be taken into account [4]; in this context, clinical protons accelerated with a cyclotron are considered the most promising tool for the near future [5].

Several explanations have been proposed to elucidate the FLASH mechanisms [6,7], but none has been able to provide a comprehensive and unique picture so far. While the initial hypotheses largely relied on a transient induced “local” hypoxia due to the oxygen depletion because of the high dose and dose rates [8], this theory called transient oxygen depletion (TOD) has been largely shown as inconsistent [9,10,11]. It is now indeed established that the origin of the FLASH effect has to be searched in a complex network of biochemical processes taking place at the cellular level during and shortly after irradiation, with oxygenation still playing a role albeit not in the simple way initially suggested, and pointing also to a multiplicity of factors, including reactive oxygen species (ROS), their recombination, iron, and lipid metabolism, which, taken together, might contribute to explaining not only the sparing effect of healthy tissues but also the concurrent iso-effectiveness of the treatment on cancer cells [7,11,12,13,14].

In this study, we used zebrafish embryos to investigate the effects of UHDR proton irradiation at two different oxygenation levels on the development process. Zebrafish share approximately 70% of human genes, and over 80% of known human disease-related genes—including oncogenes and tumor suppressors—have at least one ortholog. Combined with their high throughput and relative ease of handling, zebrafish serve as an effective intermediate model for studying biological responses [15]. This is also the case of the investigation for the mechanistic bases of the FLASH effect, being a purely in vivo effect, but presenting some features that are emerging and can be partially explorable also with in vitro assays [16]. Zebrafish, representing the ideal bridge between the two approaches, were thus previously employed extensively for the study of the FLASH effect [17,18,19,20,21,22,23,24,25]. Different endpoints and irradiation conditions were explored, and contradictory results have been reported so far. While some studies showed a significant FLASH effect on the malformation rate of multiple organs, other works did not observe significant differences, or they did so only for some specific tissues.

One important aspect that we explored is the interplay of the protective effect induced by the dose rate and the level of oxygenation. In fact, while, as above mentioned, the specific impact of oxygen in the FLASH mechanism is far from being understood, beside the disproof of the TOD, significant results reported an increased emergence of the FLASH effect at lower oxygenation conditions, with mice novel object recognition tests [17]. Such an effect was confirmed also by zebrafish experiments, with a larger occurrence in hypoxic conditions [18,19,21]. At the same time, it appeared that reactive oxygen species and radical recombination may be importantly correlated by the observed sparing effect versus dose rate [9].

We present in this work an analysis of developmental and morphological endpoints such as larval length, yolk malabsorption, pericardial edema, head and eye size, and spinal curvature, which we employed to assess the extent of normal tissue sparing under UHDR conditions. The role of hypoxia was also investigated. This approach aims to confirm and extend previous findings in zebrafish while addressing gaps in our understanding of dose-rate and oxygenation dependencies in FLASH RT.

## 2. Materials and Methods

### 2.1. Zebrafish Care and Mating

Adult zebrafish (*Danio rerio*) wild-type AB-strain were housed at the Model Organism Facility, Department of Cellular, Computational and Integrative Biology (CIBIO Department, University of Trento, Italy) and they were kept under standard conditions (14 h light/10 h dark cycle, temperature 27.5 °C, conductivity 500 ± 50 μS, and pH 7.5).

All experiments involving zebrafish were conducted in compliance with the European and Italian regulations (D.Lgs. 26/2014). No ethical approval for the use of fish was required since larvae were used before the fifth day post-fertilization. Adult fish used to generate embryos belonged to the colony of MOF authorized as breeders by the Municipality of Trento (C_L378/S022/103359/03.06.2015).

### 2.2. Preparation of Zebrafish Samples for Treatments

Three independent experimental replications were performed on different days using embryos from different breeding couples of the same line. The number of embryos for each replicate of the same experimental condition was variable in the range 23–27.

To place zebrafish embryos for irradiation, we used 0.5 mL Eppendorf tubes as support. According to a previously proposed protocol [21], we melted agarose to a concentration of 0.8–1%, and we carefully pipetted it into the conical bottom part of the Eppendorf tubes. Next, once the agarose had cooled and solidified, we gently placed 23–27 embryos at 24 h post-fertilization (hpf) onto the solid agarose layer and distributed them uniformly. The embryos occupied a final thickness of about 1.5 mm. Finally, the entire Eppendorf tube was filled with E3 medium. No methylene blue was used in the E3 medium to avoid interfering with redox reactions induced by irradiation.

Half of the prepared samples were irradiated under normoxic and half under hypoxic conditions. For the hypoxic group, the Eppendorf tubes with zebrafish embryos were placed with open caps inside a controlled hypoxic chamber (Ruskinn^®^ INVIVO2 200, Ruskinn Technology Ltd., Bridgend, UK). The chamber was adjusted to establish and maintain a low-oxygen environment (O_2_: 1%, CO_2_: 0%, T: 28 °C). Embryos were kept in the chamber for 90 min to achieve a stable hypoxic state. Tubes were closed to prevent reoxygenation before irradiation, and lids were sealed with vacuum grease and Teflon.

As verification, and to simulate the real timing of the irradiation experiment, oxygen measurements were taken at specific time points (i.e., 60, 90, and 120 min), after removal from the hypoxia chamber and sealing of the Eppendorf tubes, using the PreSens PM-PSt8 needle of PreSens Precision Sensing system (Regensburg, Germany) (Schematic representation and clos-up photo of the system in Supplement S1). Overall, after incubation in the hypoxic chamber, the measured oxygen level was equal to 1.3% ± 0.5% (range 0.8–1.8%) in the monitored time interval.

### 2.3. Monitoring of Zebrafish Development

After irradiation, embryos were transferred to 96-well plates, with one embryo per well, to monitor the development individually over time. The plates were maintained at a constant temperature of 28 °C and monitored daily, at consistent time points. Pictures of all embryos were acquired using a Leica^®^ MZ16 stereomicroscope with bright light illumination. Quantitative analyses of developmental malformations were conducted twice by two independent operators, by assessing the following features:

– Larval length [μm]
– Yolk absorption [μm]
– Pericardial edema [μm]
– Head size [μm]
– Eye size [μm]
– Spinal curvature [(180—angle)°]

**Figure 1:**
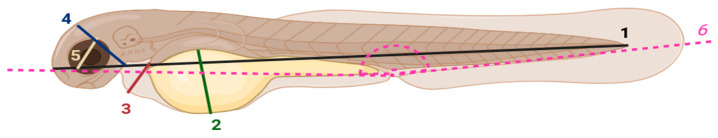
The morphological analyses/classifications performed in this study were based on the measurement of specific features associated with the anatomical sites indicated in the figure in terms of: (1) larval length—black line, (2) yolk malabsorption—green line, (3) pericardial edema—purple line, (4) head size—purple line, (5) eye size—yellow line, and (6) spinal curvature—dotted arch of dotted pink line. Image created with BioRender^®^.

Measurements were performed manually at 119 hpf using ImageJ software (ImageJ-1.54f (64bit version)), after appropriate calibration, as showed in Figure 1.

A morphological scoring system, originally developed by Brannen et al. [26] and described by Szabò et al. [27], was also adopted for the analysis of two specific endpoints, namely pericardial edema and spine curvature. Images of individual zebrafish larvae at the same time point (119 hpf) were retrospectively assessed, and visible malformations were classified according to the scoring system, as described in Supplement S2.

### 2.4. Irradiation and Dosimetry

Zebrafish embryo irradiation was performed at the Trento proton beam line (TPBL), which is part of the local proton therapy center (Azienda Provinciale per i Servizi Sanitari, APSS, Trento, Italy). The irradiation facility was extensively described in the past [28,29]; recently it introduced the possibility of performing UHDR proton irradiation. To this purpose, new beam transport optics were tuned for the highest available proton beam energy of 228 MeV, for which the highest transport efficiency can be reached. The proton beam at the irradiation position was then characterized in terms of lateral profile, intensity, and stability. Details on the beam characteristics are reported in Supplement S3 (Figure S2).

The embryos were irradiated in 0.5 mL Eppendorf tubes, upon alignment at irradiation position by means of dedicated lasers. Each tube received an expected dose of 30 Gy, delivered either at a conventional dose rate (0.6 Gy/s) or UHDR (317 Gy/s). The dose delivery was monitored online based on the counting obtained from a previously calibrated integral ionization chamber. Additionally, an EBT-XD gafchromic film (Ashland Inc., Wilmington, DE, US) was irradiated together with each sample and the delivered dose was then reconstructed offline.

### 2.5. Statistical Analysis

Concerning radiation-induced malformations, each data point in the plots represents the inverse-variance weighted average calculated for the three replicates, together with the associated standard deviation. Similarly, a mean score value was calculated for each replicate for both endpoints (i.e., pericardial edema and spine curvature). An average value was then computed accounting for the three replicates, together with the standard deviation. Statistical significance was evaluated by means of Student’s *t*-test. Statistical significance was attributed to *p*-values < 0.05.

## 3. Results

**Figure 2:**
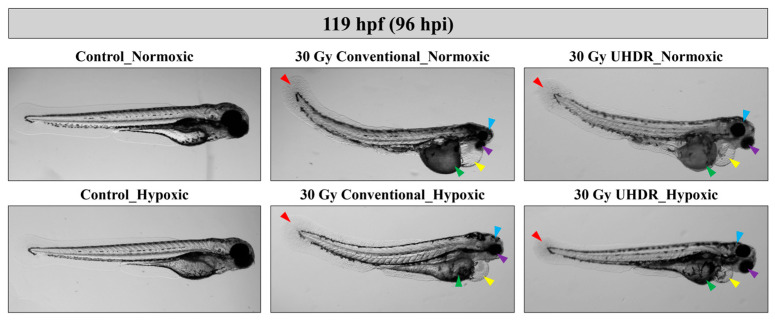
Microscopy acquisition of representative zebrafish samples at 119 hpf, in different conditions based on oxygen levels (Normoxia vs. Hypoxia on the upper and lower row, respectively) and radiation treatment (Control, Conventional, and UHDR irradiation, from left to right). The pictures show different developmental abnormalities, which are marked with colored symbols corresponding to Spinal curvature—Red Microcephalia—Blue; Microphthalmia—Purple; Pericardial edema—Yellow; Yolk malabsorption—Green.

**Figure 3:**
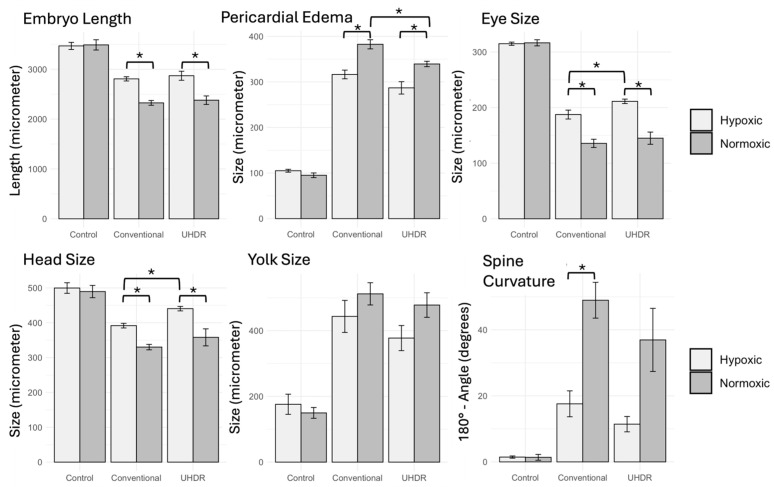
Quantitative comparisons of various morphological features under different experimental conditions. The conditions analyzed are control, conventional, and UHDR irradiation (from the left to right in each subplot, respectively), under hypoxic (white bars) and normoxic (gray bars) conditions. Measurements are in µm except for degrees in spine curvature. Statistical significance is marked by asterisks (*p* < 0.05 *), error bars indicate the standard deviation calculated over three independent experiments.

Zebrafish survival rate was monitored until 119 hpf, corresponding to about 96 h post irradiation, and no differences were found between control and irradiated samples. The morphological malformations observed on zebrafish larvae are displayed in Figure 2 for representative cases, according to the different treatment conditions. A quantitative analysis of the different endpoints is reported in Figure 3. As expected, data show a radioprotective effect associated with the hypoxic irradiation environment compared to the normoxic one. This is consistently observed for each endpoint both for zebrafish irradiated at conventional and UHDR, which is associated in most cases with statistical significance. In terms of the dose rate parameter, there is a trend of slightly reduced toxicity after exposure to UHDR, which is maintained under both normoxic and hypoxic conditions. However, the difference turns out to be significant for a limited number of cases, i.e., normoxic irradiation for pericardial edema (382 ± 10 μm conventional vs. 339 ± 6 μm UHDR, *p* = 0.01), hypoxic irradiation for eye (187 ± 8 μm conventional vs. 211 ± 4 μm UHDR, *p* = 0.03) and head size (391 ± 6 μm conventional vs. 440 ± 6 μm UHDR, *p* = 0.001).

In terms of relative variation, the pericardial edema is reduced by about 11% at UHDR, while the measured eye and head size are larger by about 13% and 12%, respectively. Notably, for pericardial edema the difference between conventional and UHDR irradiation approaches does not reach significance also for the hypoxic case (Supplement S3 Table S2 for *p*-values). Control groups under normoxic and hypoxic conditions show comparable developmental outcomes, indicating that the temporary hypoxic incubation does not adversely affect zebrafish development.

**Figure 4:**
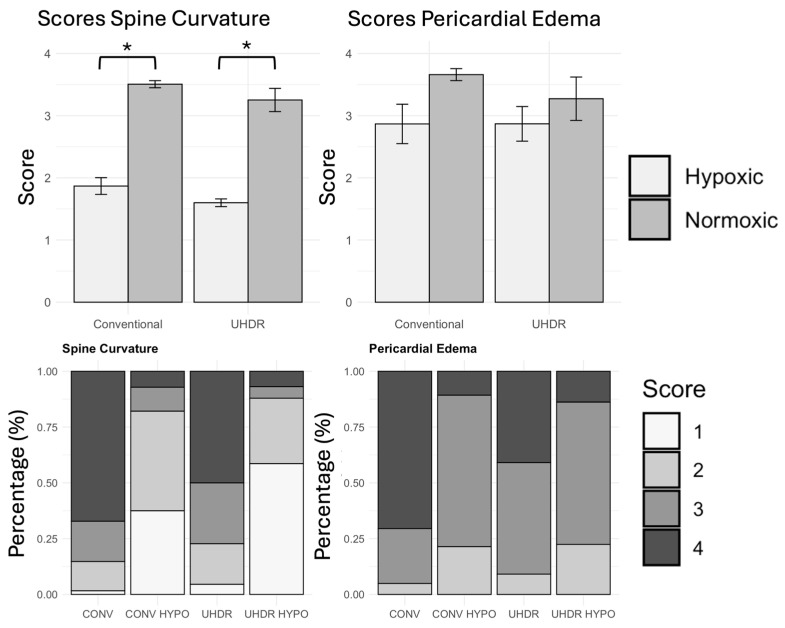
Average toxicity scores (**upper** panels) and percentage distribution (**lower** panels) for SC and PR at 119 hpf, following irradiation under normoxic and hypoxic conditions. Conditions include control conventional and UHDR irradiation. Toxicity scores were computed based on the ordinal ranking of morphological severity: 1 = no altered phenotype, 2 = mild, 3 = moderate, 4 = severe, as adapted from [26,27]. Statistical significance is marked by asterisks (*p* < 0.05 *), error bars indicate the standard deviation calculated over three independent experiments.

Similar results emerge from the analysis of the average toxicity score (i.e., SV mean) for pericardial edema and spinal curvature (Figure 4). A differential response is observed for embryos irradiated under hypoxic or normoxic conditions; however, significant differences are only attributed to spinal curvature. The possible protective role of UHDR here translates into slightly lower average scores attributed to UHDR samples, which nevertheless do not result in statistically significant deviations from conventional ones. When comparing conventional and UHDR, we observe for spinal curvature an average score reduction of approximately 7% and 15% for normoxic and hypoxic irradiation, respectively; for pericardial edema, the average score is comparable for the hypoxic samples, while a reduction of about 10% is associated with UHDR under normoxia.

These results are complemented by the scoring percentage distribution, summarized in Figure 4. On top of the obvious dependence on the oxygenation level, these data show a tendency toward a protective effect of UHDR, which is less evident when looking at the average scores (Figure 4). UHDR appears to be associated with a lower percentage of embryos in the high score categories. This is observed both for pericardial edema and spinal curvature, as well as under normoxic and hypoxic conditions, with the only exception registered for the spinal curvature endpoint for hypoxic irradiation. (Supplement S3 Table S3 for *p*-values). 

## 4. Discussion

An increasing interest in Zebrafish as an experimental model for radiobiology research has been registered in recent years. This is confirmed by several publications focused on investigating the FLASH effect [17,18,19,20,21,22,30,31], but also by other studies unrelated to UHDR and dedicated to comparing the biological effects of different radiation qualities [26,27]. Although zebrafish possess inherent limitations compared to mammalian models—most notably in terms of tissue complexity and immune system maturation—they offer significant advantages in scalability and experimental throughput. Their optical transparency, external development, and rapid embryogenesis allow for high-resolution phenotypic assessment and facilitate high-content screening approaches that are essential for dose–response characterization and multivariate analyses.

We presented the results of a comprehensive study of zebrafish developmental malformations induced by conventional and UHDR irradiation, comparing the results for normoxic and hypoxic conditions. Our study contributes to the ongoing research dedicated to the understanding of the FLASH effect, a context in which zebrafish have been employed obtaining partially contradictory results. Montay-Gruel et al. [17] showed a protective effect of UHDR electrons compared to a conventional dose rate after 8 Gy electron irradiation. Interestingly, when the samples were irradiated in the presence of antioxidants (amifostine or NAC), a sparing effect was observed after conventional exposure but not after UHDR. Beyreuther et al. were the first to report experimental outcomes on embryos irradiated with protons at different dose rates [18]. While those results showed a FLASH effect limited to a single end-point (i.e., pericardial edema), a later publication employing irradiation at controlled oxygenation (i.e., pO_2_ < 10 mmHg, obtained by sealing zebrafish embryos inside the Eppendorf tube and thus consuming oxygen without replacement) indicated a significant radioprotection at a UHDR for embryo length, spine curvature, and pericardial edema, both after electron and proton irradiation [21]. Consequently, a protocol for embryo preparation was proposed to allow irradiation under hypoxia, which we adapted to our experiments that include incubation inside a hypoxic chamber. Interestingly, the authors reported an increase in length of about 3% at a UHDR compared to conventional, which is in line with the about 2.2% increase observed in our study. Such results were further corroborated by the dose response curves published by the same group [19], employing protons at the entrance and mid-SOBP (i.e., spread-out Bragg peak). In contrast, Kacem et al. [22] reported negative results in terms of a missing radioprotective effect after either x-ray, electron or proton irradiation; remarkably, oxygenation levels were not monitored during those experiments, leaving room for the possible influence of the hypoxic environment on the FLASH effect induction. In Jansen et al. 2022 [9], oxygen depletion was measured in the same conditions of the zebrafish irradiations, and it was taken as an alias of the radical recombination rate. Larger dose rates correlated with a lower depletion, a value confirmed by several following studies, thus indicating, in addition to another point of inconsistency for the TOD hypothesis, a possible increase in radical recombination at a UHDR as a source of the protective effect. Additional results were obtained by Saade et al. [25], who adopted the protocol proposed in [21] and therefore irradiated zebrafish embryos at comparable oxygen levels. The authors showed a sparing effect for embryo length and pericardial edema, while no significant effect was associated with spinal curvature. In this case, embryo length was about 6% larger at 30 Gy UHDR compared to conventional, an effect somewhat larger than the 2.2% increase that we reported. Interestingly, the FLASH effect was more pronounced at 30 Gy than at 40 Gy, suggesting a threshold or saturation phenomenon in the biological response. In a separate publication, malformations were observed after helium ion irradiation, obtaining results overall in line with the previous study [23]. The same group recently published partially contradictory results [24], comparing proton irradiation at conventional and extremely high dose rates. In this case, no sparing was observed on morphological endpoints. However, they highlighted a higher level of apoptosis at conventional irradiation compared to UHDR, supported by higher levels of DNA fragmentation detected by the TUNEL test and higher expression levels of genes controlling cell cycle and p53-dependent apoptosis.

Our findings fit in this scenario, as samples subjected to UDHR-hypoxic conditions showed a better sparing of all tissues evaluated for toxic effects, despite not always being supported by statistical significance. Remarkably, the protective effect is observed both for normoxic (pericardial edema) and hypoxic (head and eye size) conditions. Taken together, these results suggest that while UHDR may reduce radiation-induced damage, its protective effects are endpoint-dependent. The role of oxygenation might as well be pronounced to a different extent depending on the tissue involved. The different degrees of response observed for different tissues agree with the recent report by Bogaerts et al. Combining gene expression analysis with immunohistochemistry, they were able to show that, for instance, apoptosis is not uniformly activated but is more pronounced in the tail compared to the head region. This paves the way for further studies exploiting molecular biology techniques to enhance our understanding of the differential response associated with the FLASH effect, possibly including information on oxygenation levels.

In this context, a recent modeling work of our group [14] suggested a tissue-dependent antioxidant rate as the main driver of the differential sparing effect between normal tissue and tumor, through the process of organic radical recombination. Thus, an immediate extension of the present work providing further insights would be the inclusion of specific antioxidant molecules to check how they could modulate the occurrence of the sparing in hypoxic and normoxic conditions.

In summary the obtained results evidence a dose rate-dependent protective effect which appears only at specific endpoints, thus highlighting a potential role of the tissue (chemical) environment for its manifestation. Moreover, we observe the effect at any oxygen concentration, thus pointing out the consistency of FLASH sparing also in normoxic conditions, differently from other works but similarly to what happens, e.g., for in vitro irradiations [32]. Finally, the extent of the UHDR-induced sparing is notably smaller as compared to the hypoxia-induced one, the latter being also present in all the analyzed endpoints, thus remarking the large difference in the two effects, and providing additional, indirect support to the implausibility of the RTOD hypothesis.

## 5. Conclusions

Our study indicates that, while the UHDR confirms its potential to reduce radiation-induced damage, these protective effects are importantly endpoint-dependent. While the pure effect of hypoxia-induced radioprotection appears instead ubiquitous and larger in size, in the combination of the two the relative role of oxygenation in the UHDR might as well be dependent on the tissue involved. Further developments of the present work will include gene expression studies, aiming at a better understanding of the pathways activated by the different irradiation regimes; the role of radioprotectors, and their interplay with dose rate and oxygenation, will be investigated as well, to shed additional light on the channels associated with UHDR-induced damage reduction.

## Data Availability

The raw data supporting the conclusions of this article will be made available by the authors on request.

## Supplementary Materials

The following supporting information can be downloaded at: https://www.mdpi.com/article/10.3390/cancers17152564/s1, Supplement S1, Figure S1: Description of the setup employed for the measurement of oxygenation; Supplement S2: Table S1: Description of the scoring system for the evaluation of spine curvature and pericardial edema. Supplement S3, Figure S2: Beam characterization and details on the proton irradiation setup; Supplement S4: Table S2: *p*-values associated with morphological measurements presented in Figure 3. Table S3: *p*-values associated with the scoring system data shown in Figure 4.
