# Supplementary material for: Investigating the Influence of Conventional vs. Ultra-High Dose Rate Proton Irradiation Under Normoxic or Hypoxic Conditions on Multiple Developmental Endpoints in Zebrafish Embryos
Source: Cancers (Basel). 2025 Aug 3;17(15):2564. doi: 10.3390/cancers17152564 (PMC12345739; doi:10.3390/cancers17152564)
Supplement: Supplementary file 1 [file cancers-17-02564-s001.zip › cancers-3746817-supplementary.pdf]

Supplement S1. Description of the setup employed for the measurement of oxygenation

To assess the actual oxygen concentration in E3 medium under hypoxic conditions, a dedicated measurement setup was employed (see Figure S1). Zebrafish embryos (n=23–27) at approximately 22 hours post-fertilization (hpf) were placed in 0.5 mL Eppendorf tubes containing a base layer of 200 µL of solidified 1% agarose, overlaid with 330 µL of E3 embryo medium. The tube cap was modified with a small hole to allow the insertion of the optic fiber oxygen probe (PreSens PM-PSt8 needle-type system). The opening was then sealed with vacuum grease and Teflon film to minimize exchange with the external environment.
Prior to measurement, the Eppendorf tubes were left open inside the $INVIVO_2$ 200 hypoxic chamber (Ruskinn®) in order to reach equilibrium conditions in the modified atmosphere (O_2: 1%, CO_2: 0%, T: 28°C). The tubes were then sealed inside the chamber and removed after incubation for oxygen monitoring. Measurements of oxygen concentration were conducted at 60, 90, and 120 minutes post-removal from the hypoxic environment to simulate the potential time frame between sample preparation and irradiation. Additionally, environmental conditions (i.e. air temperature, relative humidit, and absolute atmospheric pressure) were monitored using an external sensor based on the Bosch BME280 module and employed to correct the readings of the oxygen probe according to the calibration sheet provided by the manufacturer. Minor differences were observed as a function of the incubation time (minimum 60 min), and the measured oxygen concentrations within the sealed tubes after incubation ranged between 0.8% and 1.8% pO_2 during the analysed time course.

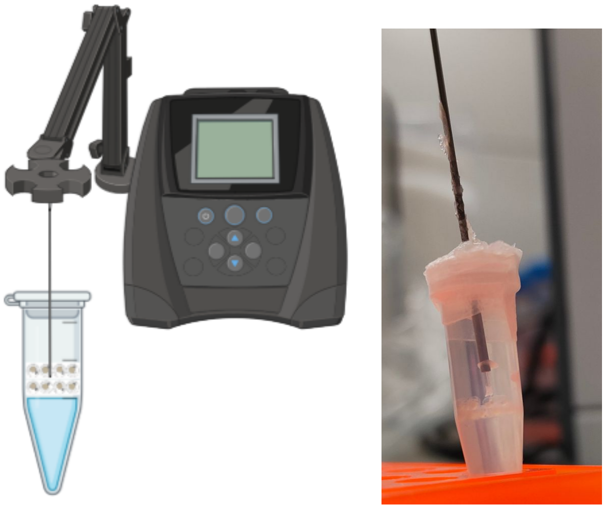

Figure S1 : Description of the setup employed for the measurement of oxygenation

Supplement S2. Description of the scoring system for the evaluation of spine curvature and pericardial edema.

The scoring system used for radiation induced morphological malformations, as reported by *Brannen KC et al., 2010* [26] and *Szabò ER et al., 2019* [27] was adopted in this study. Specifically, pictures of individual embryos at 3 dpi were manually analyzed in order to qualitatively categorize, with a value from 1 to 4 referring to toxicity level as described in Table S2, the entity of pericardial edema (PE) and spinal curvature (SC). In accordance with *Brannen KC* and *Szabò ER*, a 'mean scoring value (SV_{Mean})' describing the PE and SC average damage induced by radiation was calculated on basis of this formula, with 'N' representing the number of embryos scored for the respective category:

$$SV_{Mean} = \frac{(4 \cdot N_{score_4} + 3 \cdot N_{score_3} + 2 \cdot N_{score_2} + N_{score_1})}{N_{living\ embryos}}$$

.

Table S1 : Description of the scoring system for the evaluation of spine curvature and pericardial edema

**Malformation**	**Score**			
	**1**	**2**	**3**	**4**
**Spine Curvature (SC)**	Normal spine	Curved end of tail	Slight bending from half of the body	Most severe curvature
**Pericardial Edema (PE)**	Normal healthy state	Very small edema	Marked abnormality, with PE size smaller than the head size	Major disorder, with PE size equal or even larger than the head size

Supplement S3. Details on the proton irradiation setup.

Proton beam irradiations were performed with a 228 MeV proton beam in the experimental area of the Trento Proton Therapy Center (APSS), for which a dedicated beam transport optics was previously set up. This translates in a beam transport efficiency of approximately 76%, which allows obtaining a ultra-high dose rate (UHDR) regime for an adequate beam current request (i.e. beam current in the range 1-500 nA can be requested). The samples were irradiated at the maximum available beam energy, without using degraders

Here in the left plot we show the X and Y beam spot profiles measured in air with a scintillating screen at irradiation position (i.e. approximately 150 cm from beam pipe). The profiles are gaussian-like, even though not perfectly symmetric, with a standard deviation of about 1 cm at irradiation position. This slight loss of quality compared to the standard beam optics is due to the need to maximize transport efficiency. Samples to be irradiated were positioned at the centre of the spots, supported also by the use of gafchromic films.
The right plot reports a verification of the output linearity for increasing beam current requests. The figure shows the reading of a monitor chamber as a function of the accelerator output current, together with a linear fit resulting in good agreement with data points. This demonstrates that the actual dose rate on irradiated samples can be adjusted simply by requesting a specific beam current.

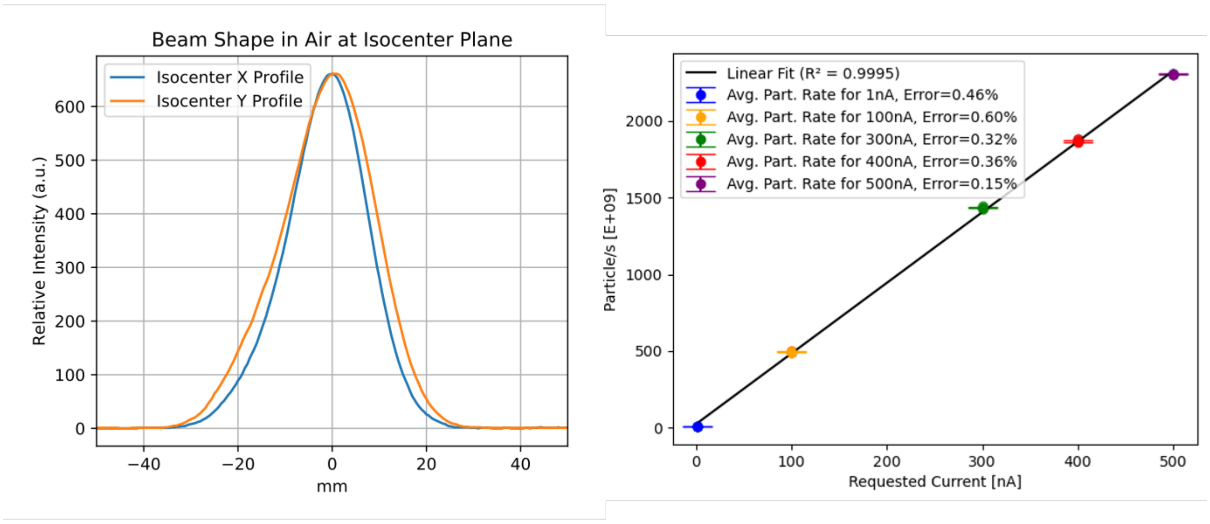

Figure S2: Beam characterization and details on the proton irradiation setup

The offline dosimetry based on EBT-XD gafchromic films revealed that the average dose delivered to samples irradiated at conventional and at ultra-high dose rate was equal to 33.6±1.8 Gy and to 33.9±1.0 Gy, respectively.

Supplement S4. Summary of p-values resulting from the statistical analysis.

Table S2： P-values associated with the data shown in Figure 3. The p-values that are considered significant (i.e. p<0.05) are in bold characters.

	**Comparison**	**p-value**
**Embryo Length**	CONV Normoxic vs UHDR Normoxic	0.4910
	CONV Normoxic vs CONV Hypoxic	**0.0005**
	UHDR Normoxic vs UHDR Hypoxic	**0.0051**
	CONV Hypoxic vs UHDR Hypoxic	0.4442
**Pericardial Edema**	CONV Normoxic vs UHDR Normoxic	**0.0109**
	CONV Normoxic vs CONV Hypoxic	**0.0025**
	UHDR Normoxic vs UHDR Hypoxic	**0.0190**
	CONV Hypoxic vs UHDR Hypoxic	0.0737
**Eye Size**	CONV Normoxic vs UHDR Normoxic	0.3813
	CONV Normoxic vs CONV Hypoxic	**0.0026**
	UHDR Normoxic vs UHDR Hypoxic	**0.0074**
	CONV Hypoxic vs UHDR Hypoxic	**0.0336**
**Head Size**	CONV Normoxic vs UHDR Normoxic	0.2425
	CONV Normoxic vs CONV Hypoxic	**0.0012**
	UHDR Normoxic vs UHDR Hypoxic	**0.0343**
	CONV Hypoxic vs UHDR Hypoxic	**0.0017**
**Yolk Size**	CONV Normoxic vs UHDR Normoxic	0.3943

	CONV Normoxic vs CONV Hypoxic	0.1856
	UHDR Normoxic vs UHDR Hypoxic	0.0563
	CONV Hypoxic vs UHDR Hypoxic	0.2108
**Spine Curvature**	CONV Normoxic vs UHDR Normoxic	0.2152
	CONV Normoxic vs CONV Hypoxic	**0.0037**
	UHDR Normoxic vs UHDR Hypoxic	0.0564
	CONV Hypoxic vs UHDR Hypoxic	0.1448

Table S3: P-values associated with the data shown in Figure 4. The p-values that are considered significant (i.e. $p<0.05$) are in bold characters.

	**Comparison**	**p-value**
**Pericardial Edema**	CONV Normoxic vs UHDR Normoxic	0.2532
	CONV Normoxic vs CONV Hypoxic	0.0608
	UHDR Normoxic vs UHDR Hypoxic	0.2734
	CONV Hypoxic vs UHDR Hypoxic	0.9964
**Spine Curvature**	CONV Normoxic vs UHDR Normoxic	0.1869
	CONV Normoxic vs CONV Hypoxic	**0.0009**
	UHDR Normoxic vs UHDR Hypoxic	**0.0032**
	CONV Hypoxic vs UHDR Hypoxic	0.0884
